# The Sbi Protein Contributes to *Staphylococcus aureus* Inflammatory Response during Systemic Infection

**DOI:** 10.1371/journal.pone.0131879

**Published:** 2015-06-30

**Authors:** Cintia Daniela Gonzalez, Camila Ledo, Constanza Giai, Ailin Garófalo, Marisa I. Gómez

**Affiliations:** 1 Instituto de Investigaciones en Microbiología y Parasitología Médica (IMPaM), Universidad de Buenos Aires, Consejo Nacional de Investigaciones Científicas y Técnicas, Buenos Aires, Argentina; 2 Departamento de Microbiología, Parasitología e Immunología, Facultad de Medicina, Universidad de Buenos Aires, Buenos Aires, Argentina; French National Centre for Scientific Research, FRANCE

## Abstract

*Staphylococcus aureus* is an important human pathogen that causes infections that may present high morbidity and mortality. Among its many virulence factors protein A (SpA) and Staphylococcal binding immunoglobulin protein (Sbi) bind the Fc portion of IgG interfering with opsonophagocytosis. We have previously demonstrated that SpA interacts with the TNF-α receptor (TNFR) 1 through each of the five IgG binding domains and induces the production of pro-inflammatory cytokines and chemokines. The IgG binding domains of Sbi are homologous to those of SpA, which allow us to hypothesize that Sbi might also have a role in the inflammatory response induced by *S*. *aureus*. We demonstrate that Sbi is a novel factor that participates in the induction of the inflammatory response during staphylococcal infections via TNFR1 and EGFR mediated signaling as well as downstream MAPKs. The expression of Sbi significantly contributed to IL-6 production and modulated CXCL-1 expression as well as neutrophil recruitment to the site of infection, thus demonstrating for the first time its relevance as a pro-inflammatory staphylococcal antigen in an *in vivo* model.

## Introduction

*Staphylococcus aureus* is a human pathogen that causes a wide variety of infections which in certain cases may have high morbidity and mortality [1]. In addition to the tissue damage that *S*. *aureus* toxins can cause in the host [1], staphylococcal infections are characterized by a profound inflammatory response and very often the inflammation may account for the pathological consequences of infection as it is the case of pneumonia, sepsis and other invasive diseases [2]. Among the bacterial compounds that induce inflammation in the host are teichoic acids and peptidoglycan which trigger pro-inflammatory signaling via recognition by TLR2 and NOD receptors, respectively [3]. More recently, it has been demonstrated that protein A induces pro-inflammatory signaling in airway epithelial and immune cells by interacting with the TNF-α receptor 1 (TNFR1) and the epidermal growth factor receptor (EGFR) and activating mitogen activated kinases (MAPKs) and nuclear factor κB (NF-κB) [4,5]. The interaction between protein A and TNFR1 which involves the five conserved IgG binding domains [6] is critical for the development of pneumonia [4].

**Fig 1:**
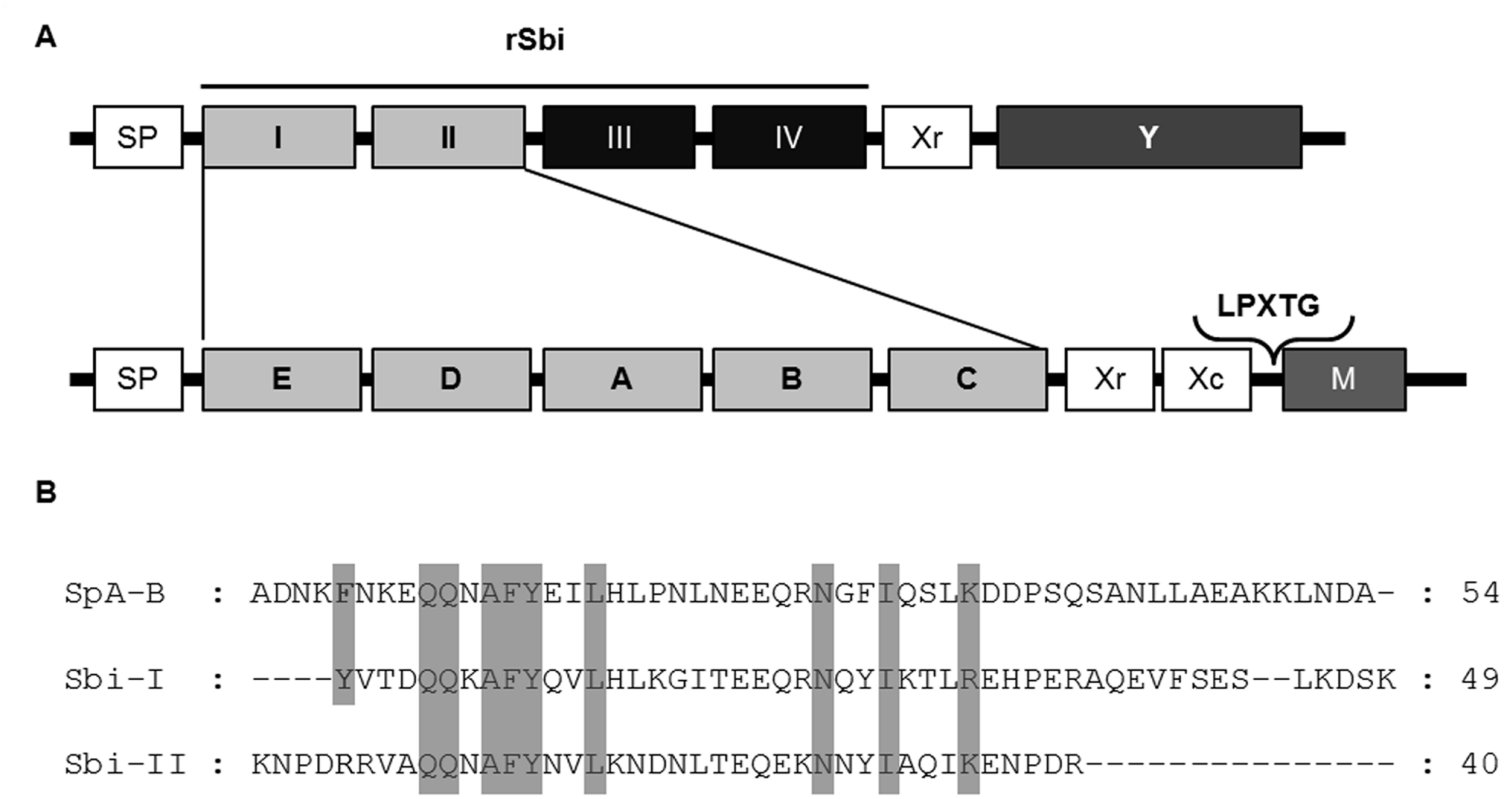
Comparison of Sbi and SpA. (A) Schematic representation of Sbi and SpA domains. The IgG binding domains (grey boxes) and the complement binding domains (black boxes) are shown. The recombinant protein Sbi (rSbi) comprising the domains I to IV was used in this study. (B) Sequence alignment of the SpA domain B and the domains I and II of Sbi. The amino acids required for SpA-TNFR1 recognition are shown in grey.

The tremendous success of *S*. *aureus* as a pathogen is due in part to its ability to evade the immune system through a variety of mechanisms [7–12]. Recently, the second binding protein for immunoglobulins (Sbi) has been described as a novel evasion factor that interferes with opsonophagocytosis and complement activity [13–16]. Sbi comprises four N-terminal globular domains (Fig 1A). The first two N-terminal domains (I and II) are homologous to the IgG binding domains of protein A and bind IgG of several species [15] whereas domains 3 and 4 (III and IV) which are independently folded interfere with the complement system [16]. Sbi can be found in association with the cell envelope, where only the domains I and II are exposed to the extracellular media, and also extracellularly as a secreted protein. Both forms of the protein contribute to immune evasion by interacting with IgG whereas only the secreted form binds complement factor C3 [17].

The IgG binding domains I and II of Sbi have high amino acid sequence identity with each of the corresponding domains of protein A. In particular, the amino acids involved in IgG recognition are identical or conserved (Fig 1B) [15]. Since these amino acids are required for protein A-TNFR1 and protein A-EGFR recognition [5,6], it is possible to hypothesize that Sbi may also interact with these cellular receptors and contribute to the induction of inflammation. Thus, this study was aimed at investigating the potential role of Sbi in the induction of inflammatory responses during *S*. *aureus* systemic infections.

## Materials and Methods

### Bacterial strains and growth

*S*. *aureus* (pCU1) strain Newman, the isogenic Sbi deficient mutant (Sbi-(pCU1)) [18], the Sbi- mutant carrying a vector that restores the expression of Sbi (Sbi-(pCU1-*sbi*)) (constructed in this work) and the isogenic SpA deficient mutant (SpA-) [18], were grown on tryptone soy agar (TSA) or tryptone soy broth (TSB) at 37°C with agitation. The levels of Sbi expression in the complemented strain were equivalent to those found in the wild type strain as compared by RT-PCR. Antibiotics (Sigma) were added as required: chloramphenicol (Cm, 10μg/ml) (to ensure maintenance of pCU1 plasmid), erythromycin (Em, 5 μg/ml) and kanamycin (Km, 50 μg/ml). For *in vivo* experiments bacteria were grown at 37°C with agitation until an OD_600_ of 0.8 washed and suspended in phosphate buffer (PBS). The medium generation time was equivalent among the wild type, the Sbi- mutant and the complemented strain. *Escherichia coli* BL21(DE3) was grown in Luria Bertani broth (LB) with Km at 37°C with agitation.

### Cloning and expression of recombinant Sbi construct

The recombinant fragment of the N-terminal region of Sbi (amino acids 28–266) comprising domains I, II, III and IV (Fig 1A), was engineered using genomic DNA from *S*. *aureus* strain Newman as template. The oligonucleotides used were: 5´-ATGCAGGGATCCAAAGCGAGTGAAAACACGCAACAAAC-3´ and 5´-AGGAGCCTCGAGTTATTACGCCACTTTCTTTTCAGC-3´. The resulting amplified fragment was subsequently cloned into the pET-28a (+) vector (using the incorporated BamHI and XhoI restriction sites). The presence of the cloned fragment was confirmed by sequencing (Macrogen). The Sbi construct was expressed in *E*. *coli* strain BL21 (DE3). Freshly transformed *E*. *coli* cells were grown until they reached an OD_600_ of 0.6. Isopropyl *β*-D-thiogalactopyranoside (SIGMA) was added to a final concentration of 1 mM and the cells were incubated at 37°C for an additional 4 h. Cells were harvested by centrifugation, suspended in binding buffer (20 mM Tris-HCl, 0.5 M NaCl, 10 mM imidazole, pH 8.0), and lysed by sonication. The lysate was centrifuged at 13000 rpm for 15 min and the supernatant was recovered and stored at -80°C. The protein was purified using nickel-ion chelating chromatography by applying supernatant to a Ni-NTA agarose resin according the manufacturer instructions (Invitrogen). The purified protein was dialyzed against PBS (Gibco) and stored at -80°C. Potentially remaining traces of lipopolysaccharide (LPS) were removed using Detoxi-Gel endotoxin-removing gel and columns (Pierce, Holmdel, NJ). The proteins were proved to be free of LPS by testing of their stimulatory capacities in the presence or absence of polymyxin B. Protein concentration was determined using a Bradford protein assay (Bio-Rad). The resulting Mw including the 34-residue N-terminal tag (MGSSHHHHHHSSGLVPRGSHMASMTGGQQMGR GS) was 31311.68 Da as determined using Compute pI/Mw tool (http://web.expasy.org/compute_pi/). The protein size was confirmed by SDS-PAGE.

### Animals and housing

Mice were obtained from the animal facility of the Department of Microbiology, School of Medicine, University of Buenos Aires. All the procedures involving laboratory animals were approved by the Institutional Committee for Use and Laboratory Animal Care (CICUAL) of the School of Medicine, University of Buenos Aires (Approval number 1101) and followed internationally accepted guidelines [19]. Animals were maintained in a conventional facility, with controlled temperature (22 ± 1°C), controlled humidity (55%), a 12:12 hour light/dark cycle and they were fed ad libitum. Procedures were performed in an experimental room within the mouse facility. Mice were euthanized using CO_2_. The number of mice required for each experiment was determined based on preliminary experiments and the desired statistical significance. The weight of the mice used was in accordance with their age. They showed good mobility and no differences in behavior were observed after manipulations. In this work molecular markers were evaluated in live animals and postmortem. The levels of pro-inflammatory cytokines in plasma from naïve mice were in the range of expected basal levels (IL-6: 0–50 pg/ml; TNF-α: 0–100 pg/ml).

### Primary Cultures

Peritoneal macrophages from BALB/c, C57BL/6 or TNFR1 deficient (*tnfr1* ^-/-^) mice (8 to 10 weeks old, 21–23 grams) were obtained by lavaging the peritoneal cavity with RPMI1640 medium containing 10% fetal bovine serum and Penicillin (100 U/ml), Streptomycin (100 μg/ml) and L-glutamine (2 mM) and plated in 96 well plates (4x10^5^ cells/well) or 6 well plates (1x10^6^ cells/well) under the same conditions. Adherent cells were selected after 3 hours by changing the media twice. Cells were grown for additional 20 hours prior to stimulation with different doses of Sbi (5 and 10 μM, corresponding to 0,156 and 0.313 mg/ml). The effect of kinase inhibitors was evaluated by pre-treating the cells with 10 μM PD98059 (MEK inhibitor V, Calbiochem), 20 μM SB202190 (p38 MAP Kinase inhibitor II, Calbiochem) or 10 μM AG1478 (EGFR tyrosine kinase inhibitor, Calbiochem) for 60 min and adding fresh inhibitors during stimulation.

### Mouse model

Female BALB/c mice (6 weeks old, 18–20 grams) were intraperitoneally inoculated with 200 μl of the Sbi recombinant protein (0,075 mg/g of mice), *S*. *aureus* (pCU1), the Sbi- deficient mutant (Sbi-pCU1) or the complemented strain (Sbi-(pCU1-*sbi*)) (4x10^7^ CFU/mice). Blood was obtained from the retro orbital sinus. Peritoneal infiltrates were obtained for cell characterization by flow cytometry and RNA extraction. For each *in vivo* determination 2–4 independent experiments with small randomly chosen groups (control and experimental groups) of 3 to 6 animals were performed to reach the final n that is specified on each figure.

### Real-time Polymerase Chain Reaction

RNA was isolated using TRIzol Reagent (Invitrogen). Complementary DNA (cDNA) was made from 1 μg of RNA using M-MLV Reverse Transcriptase (Promega). Primers and annealing temperatures used for quantitative real-time polymerase chain reaction are listed in Table 1. Glyceraldehyde 3-phosphate dehydrogenase (mGAPDH) was used as control for standardization.

**Table 1:** Primers and Annealing Temperature.

Gene	°C	Sequence (Fw;Rev)
CXCL-1	56	5′-CCGCGCCTATCGCCAATGAGCTGCGC-3′; 5’-CTTGGGGACACCTTTTAGCATCTTTTGG-3'
CXCL-10	63	5′-CTCTCGCAAGGACGGTCCGC-3′; 5′-CGTGGGCAGGATAGGCTCGG-3′
GAPDH	60	5′-GAAGGTGGTGAAGCAGGCAT-3′; 5′-TCGAAGGTGGAAGAGTGGGA-3′

### ELISA

IL-1β, IL-6 and TNF-α were quantified in culture supernatant and mouse serum by enzyme-linked immunosorbent assay using matched antibody pairs (BD Biosciences).

### Flow cytometry

Cells were stained with phycoerythrin-labeled anti-CD45 and fluorescein isothiocyanate-labeled anti-Ly6G (BD Pharmingen), washed, fixed in 1% paraformaldehyde and analyzed with a Becton Dickinson FACS Calibur. Data were collected using Cell Quest software and analyzed with Winmdi.

### Western Blot

Cells were lysed using modified RIPA buffer (50 mM Tris–HCl, 150 mM NaCl, 1 mM EDTA, 1% Triton-X-100, 1mM PMSF, 0.1% SDS, pH 7.4) containing a protease inhibitor cocktail, 1 mM sodium orthovanadate, and 1 mM sodium fluoride (Sigma-Aldrich, St. Louis, MO). Samples were incubated for 30 minutes in agitation at 4°C, centrifuged at 13000 rpm for 10 min and the supernatant collected and stored at -80°C. Proteins were separated on 8% bis-acrylamide gels, transferred to a nitrocellulose membrane, and blocked with 5% milk in TBST (50mMTris (pH 7.5), 150 mM NaCl, and 0.05% Tween) for 1 h at room temperature. Immunodetection was performed using antibodies against phosphorylated extracellular signal- regulated kinases 1 and 2 (phospho-erk1/2) (Santa Cruz Biotechnology, Dallas, TX), anti-phospho-p38 (Santa Cruz Biotechnology,Dallas, TX) or actin (Sigma-Aldrich, St. Louis, MO), followed by secondary antibodies conjugated to horseradish peroxidase (Santa Cruz Biotechnology, Dallas, TX). To evaluate the expression of the protein Sbi a protein A deficient mutant of *S*. *aureus* (SpA-) was grown in TSB until an OD_600_ of 0.5, normal mouse serum was added to a final concentration of 10% V/V and the culture was grown for 2 additional hours. Independent cultures grown without serum were used as control. Bacteria were washed with PBS, suspended in sample buffer and heated at 95°C during 10 minutes. Immunodetection was performed by overnight incubation with a donkey anti-goat IgG conjugated to horseradish peroxidase (Santa Cruz, sc-2020). Images were analyzed with ImageJ software.

### Statistics

When comparing two groups of data Student *t* Test or the nonparametric Mann Whitney test were used based on the data distribution. When comparing more than two groups of data, parametric ANOVA and Bonferroni’s multiple comparisons post-test or Kruskal-Wallis test and a Dunn’s post-test were used according to the data distribution. For the analysis of cytokine levels in serum, Wilcoxon matched-pairs signed rank test was used. For *in vivo* studies data of all the mice included in the experiments were included in the analysis. GraphPad Prism software was used for statistical analysis.

## Results

### Sbi induces pro-inflammatory cytokines in macrophages via TNFR1 and EGFR signaling

**Fig 2:**
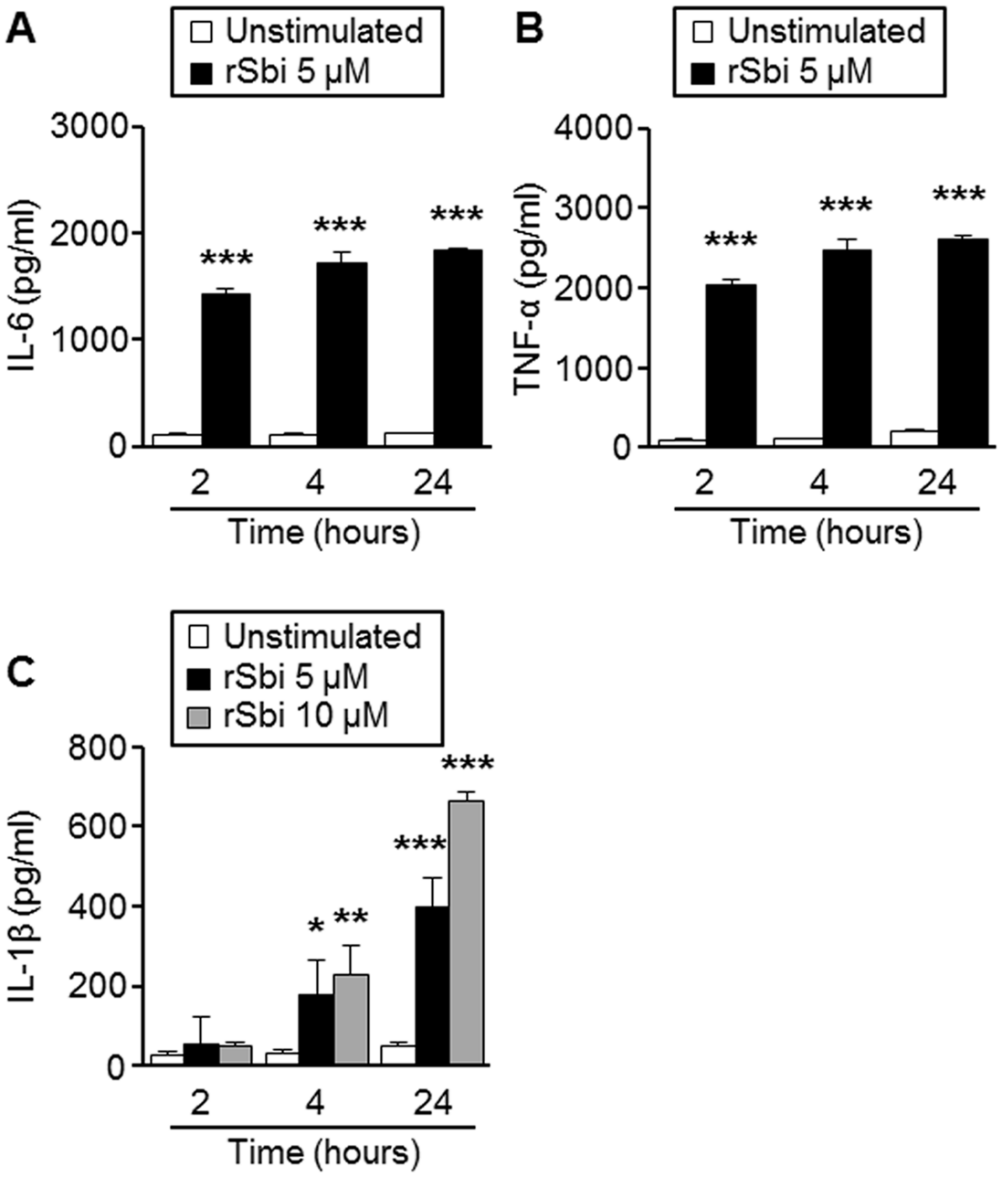
Induction of pro-inflammatory cytokines in response to Sbi. Peritoneal macrophages obtained from BALB/c mice were stimulated with the recombinant protein Sbi at the concentrations indicated for different periods of time. The levels of IL-6 (A), TNF-α (B) and IL-1β (C) were quantified in the culture supernatant by ELISA. *, *P*<0,05; **, *P*<0,01, ***, *P*<0,001; Student *t* Test.

**Fig 3:**
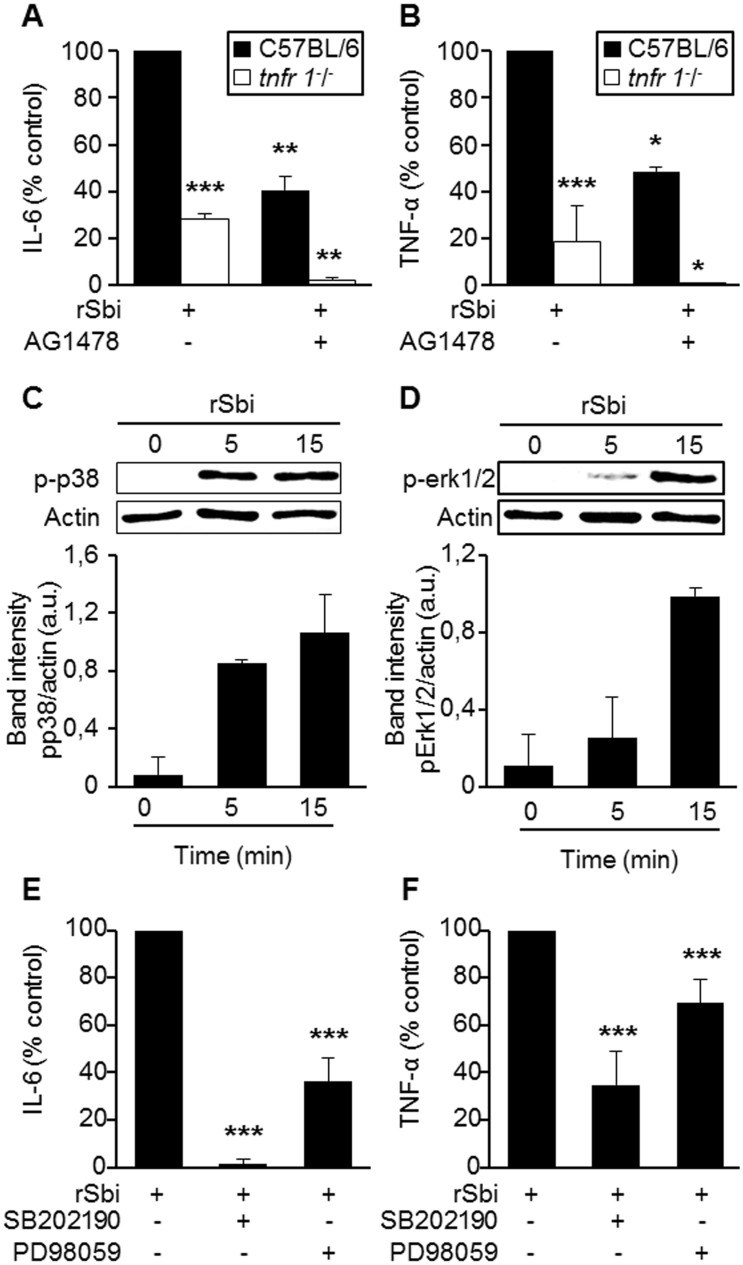
Signaling cascades induced in response to Sbi. (A and B) Peritoneal macrophages from C57BL6 or *tnfr1* ^*-/-*^mice were stimulated with the recombinant protein rSbi (5 μM) in the presence or absence of AG1478, a specific EGFR phosphorylation inhibitor. (C and D) Cells were stimulated with rSbi and p-erk1/2 and p-p38 were detected at different time points by immunoblotting. Bars show the media of the densitometric quantification of the phosphorylated species relative to actin, which was used to normalize the protein charge, from two independent experiments. a.u., arbitrary units. (E and F) Peritoneal macrophages from C57BL6 were stimulated with the recombinant protein rSbi in the presence or absence of specific chemical inhibitors for p38 (SB202190) or erk1/2 (PD98059) and the levels of IL-6 and TNF-α were quantified at two hours after the stimulation in the culture supernatant by ELISA. Each bar represents cumulative data from 3 independent experiments. *, *P*<0,05; **, *P*<0,001; ***, *P*<0,0001; Student *t* Test.

Peritoneal macrophages from BALB/c mice were stimulated with the recombinant fragment of Sbi corresponding to domains I to IV (rSbi) for different periods of time. A significant increase in IL-6 and TNF-α production was observed at 2 hours after stimulation (Fig 2A and 2B). IL-1β induction was observed after 4 hours of stimulation and further increased 20 hours later (Fig 2C). Cytokine production was not detected at earlier time points (data not shown). In order to evaluate the potential role of TNFR1 in the initial cytokine response, peritoneal macrophages from C57BL/6 or *tnfr1* ^*-/-*^mice were stimulated with rSbi. A significant decrease in both IL-6 and TNF-α production was observed in cells from *tnfr1* ^*-/-*^ mice compared with their wild type controls (Fig 3A and 3B). The addition of AG1478, an inhibitor of EGFR phosphorylation, significantly decreased the levels of both cytokines in cells from C57BL/6 mice (Fig 3A and 3B). Moreover, cytokine production was completely inhibited in the absence of TNFR1 and EGFR signaling as evidenced in cells from *tnfr1* ^*-/-*^ mice stimulated in the presence of AG1478 (Fig 3A and 3B).

The signaling cascade downstream of TNFR1 and EGFR could involve the activation of MAPKs. Thus, we next determined the ability of rSbi to activate MAPKs in macrophages. The protein induced phosphorylation of p38 and erk1/2, which are known to positively modulate the transcription of inflammatory cytokines, in macrophages starting at 5 min after stimulation (Fig 3C and 3D). Phosphorylation was transient and it was not observed by 60 min of stimulation (data not shown). In order to determine the importance of p-38 and erk1/2 phosphorylation in mediating the production of inflammatory cytokines in response to Sbi, peritoneal macrophages obtained from C57BL/6 were stimulated with rSbi in the presence or absence of specific inhibitors. The production of IL-6 was abolished in the absence of p-38 activation (Fig 3E), highlighting the importance of this MAPK in the induction of IL-6 in response to rSbi. In addition, the erk1/2 inhibitor partially blocked the production of IL-6 indicating that this MAPK is also involved (Fig 3E). Both, the p38 and the erk1/2 inhibitors partially inhibited TNF-α induction in response to rSbi (Fig 3F). These results suggest that early induction of inflammatory mediators by rSbi is mediated by TNFR1 and EGFR signaling cascades and that the MAPKs p38 and erk1/2 are involved in this response. Similar results were obtained using peritoneal macrophages from wild type BALB/c mice (data not shown).

### Sbi induces inflammatory responses *in vivo*

**Fig 4:**
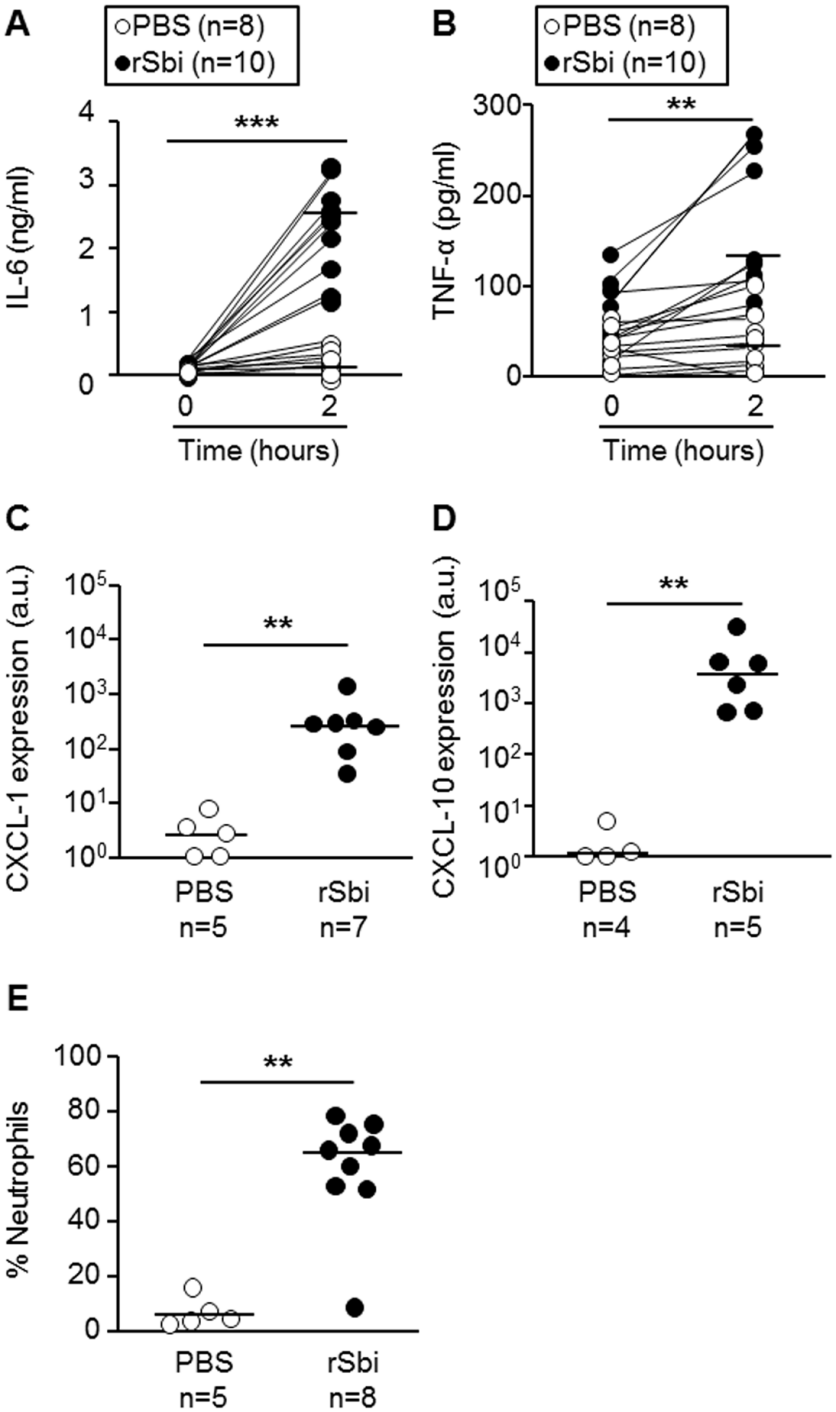
Induction of inflammatory response by Sbi *in vivo*. Groups of BALB/c mice were inoculated by intraperitoneal route with PBS or rSbi (0,075 mg/g of mice). (A and B) Two hours post-inoculation, the levels of IL–6 and TNF-α in plasma were quantified by ELISA and compared with baseline levels. **, *P*<0,01; ***, *P*<0,001, Wilcoxon matched–pairs signed rank test. (C and D) Four hours post-inoculation the expression of CXCL-1 and CXCL-10 was determined in cells recovered from peritoneal infiltrates and standardized to GAPDH expression. (E) The percentage of neutrophils recruited to the peritoneum was determined by flow cytometry. Each circle represents a single mouse (A, B and E) or a pool of two mice (C and D) and horizontal lines depict the median for each group. **, *P*< 0,01; Mann Whitney non parametric test.

We next evaluated the responses evoked by Sbi *in vivo* using a mouse model of peritoneal inflammation. Mice were inoculated with purified rSbi (0,075 mg of protein/g of mice) by the intraperitoneal route and the plasma levels of IL-1β, IL-6 and TNF-α were determined prior inoculation and 2 and 4 hours thereafter. A significant increase in the circulating levels of IL-6 and TNF-α was observed at 2 hours after inoculation (Fig 4A and 4B) and both cytokines returned to basal levels 2 hours later (data not shown). There were no significant differences in the levels of IL-1β in plasma at all the times analyzed (data not shown). A significant increase in the induction of the chemokines CXCL-1 and CXCL-10 was observed in mice inoculated with rSbi compared with those inoculated with PBS (Fig 4C and 4D). These findings correlated with a significant increase in the percentage of neutrophils recruited to the peritoneum 4 hours after the inoculation with rSbi (Fig 4E).

**Fig 5:**
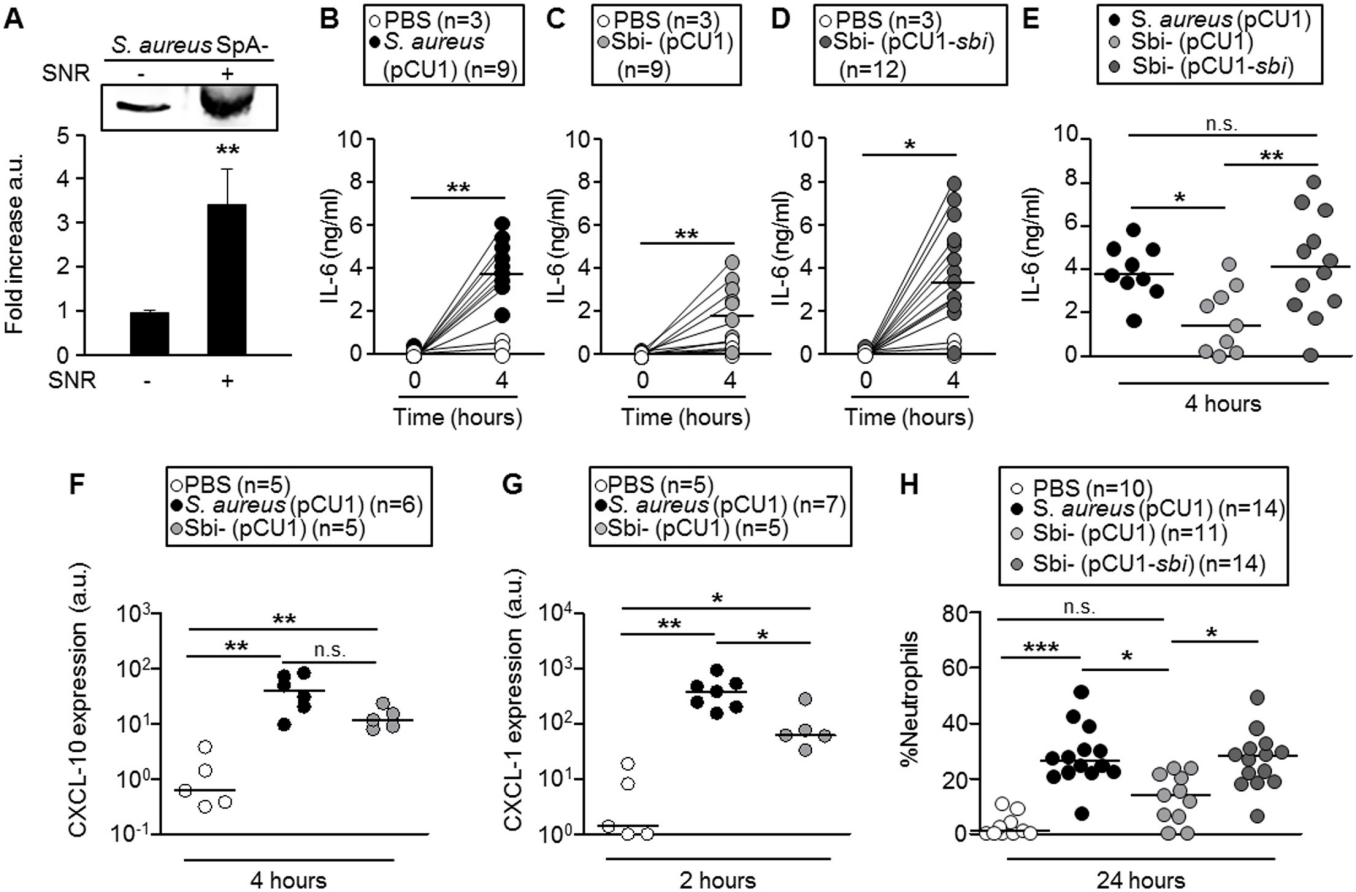
Contribution of Sbi to the inflammatory response induced by *S*. *aureus* during systemic infection. (A) Induction of Sbi expression in the presence of normal mouse serum (NMS). Bars represent the media and standard deviation from three independent experiments. *, *P*<0.05; Student *t* test. (B-H) Groups of BALB/c mice were inoculated by intraperitoneal route with PBS o 4x10^7^ CFU of *S*. *aureus* (*S*. *aureus* (pCU1)), the Sbi deficient mutant (Sbi- (pCU1)) or the complemented strain (Sbi- (pCU1-*sbi*)). (B, C and D) Four hours after the inoculation the levels of IL-6 in plasma were quantified by ELISA and compared with baseline levels. (E) Bars represent the median for each group. (F-G) The expression of CXCL-10 and CXCL-1 was determined in cells recovered from peritoneal infiltrates and standardized to GAPDH expression. (G) Twenty four hours post-inoculation the percentage of neutrophils recruited to the peritoneum was determined by flow cytometry. Each circle represents a single mouse (A, B, C, D and G) or a pool of two mice (E and F) and the horizontal lines depict the median for each group. **, *P*< 0,01; Wilcoxon matched-pairs signed rank test (A, B and C). *, *P*< 0,05; parametric ANOVA with Bonferroni’s multiple comparison post-test (D). *, *P*< 0,05; **, *P*< 0,01; Mann Whitney non parametric test (E and F). *, *P*< 0,05; ***, *P*< 0,001 non parametric Kruskal-Wallis with Dunn’s post-test (G).

We then determined the biological relevance of the above described results by using a sub-lethal model of systemic infection. It has been demonstrated that Sbi expression is up-regulated in the presence of human IgG [20,21]. In order to determine if this type of regulation would occur in mice, a SpA deficient strain (SpA-) was grown *in vitro* in the presence of mouse serum. A significant increase in the expression of Sbi was observed (Fig 5A) suggesting that during *in vivo* mouse infection the regulation of Sbi expression by serum may occur similarly to what it has been proposed for humans. A significant increase in the circulating levels of IL-6 was observed 4 hours after inoculation with wild type *S*. *aureus* (Fig 5B and 5E) whereas the levels of this cytokine were significantly lower in plasma from mice inoculated with the Sbi-mutant (Fig 5C and 5E). Mice inoculated with the Sbi complemented strain showed levels of plasmatic IL-6 equivalent to those found in mice inoculated with the wild type strain, verifying the importance of Sbi expression in the induction of IL-6 (Fig 5D and 5E). IL-1β and TNF-α were not detected at this time point in *S*. *aureus* inoculated mice as previously described for this model of infection ([22] and unpublished observations). The levels of CXCL-10, significantly increased at four hours after inoculation with *S*. *aureus* (Fig 5F). CXCL-10 was also induced in mice inoculated with the Sbi- mutant, although the levels were lower than those observed in mice inoculated with wild type *S*. *aureus* (Fig 5G). A significant increase in the expression of CXCL-1 mRNA in peritoneal infiltrates was observed at two hours after inoculation with *S*. *aureus* (Fig 5F). Although CXCL-1 was also induced at two hours after inoculation with the Sbi- mutant, its expression was significantly lower than that observed in mice inoculated with *S*. *aureus* (Fig 5G). The differential levels of chemokine induction in the peritoneum evoked by the different strains correlated with significantly decreased recruitment of neutrophils in response to the Sbi mutant compared with the response observed in mice inoculated with wild type *S*. *aureus* or the Sbi complemented strain (Fig 5H) demonstrating the importance of Sbi in the induction of inflammatory responses *in vivo*.

## Discussion

*S*. *aureus* local infections are characterized by the presence of an intense inflammatory infiltrate which may often lead to tissue damage. Similarly, the systemic inflammatory response that is induced during *S*. *aureus* infections may conduce to the development of sepsis and shock, a major cause of mortality. *S*. *aureus* harbors numerous components that elicit inflammatory signaling cascades. In the present study, using recombinant protein as well as a set of isogenic wild type, Sbi deficient and Sbi complemented strains, we demonstrated that Sbi is involved in the production of pro-inflammatory cytokines and chemokines by macrophages as well as the induction of neutrophil recruitment during systemic *S*. *aureus* infection.

Several receptors, including TLR2/TLR1, TLR2/TLR6, CD36, Nod proteins and TNFR1 have been implied in host recognition of structural components of *S*. *aureus* that elicit inflammation [3]. Our *in vitro* studies using cells from mice deficient in the expression of TNFR1 or chemical inhibition of EGFR suggest that, similar to host recognition of protein A, signaling through these receptors is required for the induction of IL-6 and TNF-α by Sbi. Consistent with TNFR1 and EGFR mediated signaling, the activation of p-38 and erk1/2 were involved in cytokine induction. The identity of the amino acids involved in protein A-TNFR1 found in Sbi suggest that this protein may directly interact with TNR1 and EGFR as previously demonstrated for protein A [4–6]. However, further studies will be required to determine whether there is a direct interaction between Sbi and TNFR1 as well as Sbi and EGFR or other accessory molecules are involved. It is also interesting to note that although certain levels of protein A can be found in the extracellular media the protein is mostly anchored to the cell wall [23]. Sbi, however, lacks the typical LPXTG motif of cell wall anchored gram-positive proteins [24] and it is found both associated to the cell membrane through interaction with lipoteichoic acids as well as secreted into the extracellular media [17]. This would allow the protein to act on cells that are not necessarily in close proximity to the bacteria. In fact, although protein A and Sbi seem to exploit similar signaling cascades to induce inflammation, the magnitude and the pattern of the cytokine induced are not exactly the same, with Sbi inducing a much more robust IL-6 production and a decreased induction of IL-1β than protein A in macrophages (unpublished observations).

In addition to the effect of Sbi on *in vitro* cultured macrophages, it significantly contributed to the elevated levels of IL-6 detected in serum during systemic *S*. *aureus* infection. The role of IL-6 during systemic bacterial infections has been controversial with studies demonstrating that this cytokine may play either protective or deleterious roles. Using the cecal ligation puncture model of peritonitis, it has been postulated that high levels of IL-6 in the early stages of infection are a marker for a bad prognosis for the outcome of the disease [25,26]. In this regard, inhibition of IL-6 has showed some beneficial effects [27] although the results varied depending on the concentration of the neutralizing antibody used [28]. However, studies using IL-6 knockout mice demonstrated that a complete lack of IL-6 strongly compromised mice in several models of infection leading to increased tissue damage and the accumulation of viable bacteria [29–32]. More recent work has clarified the dual role of IL-6 demonstrating that the cis-signaling through the membrane associated IL-6 receptor is associated with the protective and anti-inflammatory functions of the cytokine whereas the trans-signaling through the soluble form of the receptor accomplished all the pro-inflammatory effects that lead to tissue damage [33–38]. We have previously demonstrated a role for protein A in the induction of ADAM-17 activation and IL-6R shedding [39]. In this sense, protein A and Sbi could act synergistically to enhance IL-6 tran-signalling. Thus, even though IL-6 is important for the resolution of the infection, elevated levels of this cytokine are associated to exacerbated inflammatory responses, tissue damage and organ failure. In this regard, it has been recently reported that TLR2 deficiency leads to increased levels of hepatic and plasmatic IL-6 due to deregulation of IL-10 signaling and that this pro-inflammatory phenotype was associated to the increased mortality observed in TLR2 knock-out mice after systemic *S*. *aureus* challenge [40] highlighting the deleterious role of IL-6.

Sbi expression is highly regulated at different levels. It is negatively regulated by *agr* [14] and the small RNA SprD [41] and positively regulated by *sae*R/S [42] and by the presence of human IgG [20]. In the current study we demonstrated that the expression of Sbi is also increased in the presence of mouse serum. The regulation of Sbi by serum components suggests that this protein may be critically important during the early induction of inflammatory mediators after the entry of the bacteria to the bloodstream. In agreement with our findings, the *sae*R/S deletion mutant induces lower levels of pro-inflammatory cytokines as compared with the parental wild type strain in a model of peritoneal *S*. *aureus* infection [43].

It is well recognized that neutrophils are required to clear *S*. *aureus*. Nevertheless, excessive recruitment and activation of these cells may have deleterious consequences to the host. In the present study we demonstrate that Sbi, in addition to the induction of IL-6 also has a significant impact in the induction of CXCL-1 at early stages during *S*. *aureus* infection as well as in the recruitment of neutrophils to the peritoneum. Therefore, the positive contribution of Sbi to IL-6 production and to the recruitment of neutrophils could play a deleterious role during *S*. *aureus* infections and be a critical factor in the host outcome.

Much of the success of *S*. *aureus* as a human pathogen is due to its great ability to evoke an exacerbated inflammatory response but also to have multiple mechanisms to evade the immune system. As a result, the host suffers from the deleterious effects of inflammation with poor eradication of the bacteria. The role of Sbi during immune evasion has already been proposed [16,44]. In this work we demonstrate for the first time its relevance as a pro-inflammatory staphylococcal antigen suggesting that Sbi is implicated in both induction of inflammation and immune evasion.

## Supporting Information

S1 File Arrive guidelines checklist. (DOCX)
