# Supplementary material for: The Sbi Protein Contributes to Staphylococcus aureus Inflammatory Response during Systemic Infection
Source: PLoS One. 2015 Jun 30;10(6):e0131879. doi: 10.1371/journal.pone.0131879 (PMC4488394; doi:10.1371/journal.pone.0131879)

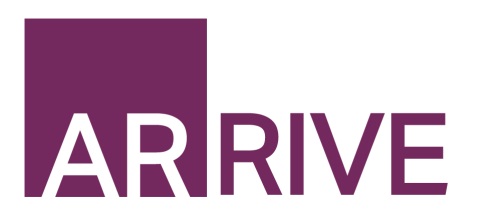


The ARRIVE Guidelines Checklist

Animal Research: Reporting In Vivo Experiments

Carol Kilkenny^1^, William J Browne^2^, Innes C Cuthill^3^, Michael Emerson^4^ and Douglas G Altman^5^

*^1^The National Centre for the Replacement, Refinement and Reduction of Animals in Research, London, UK, ^2^School of Veterinary Science, University of Bristol, Bristol, UK, ^3^School of Biological Sciences, University of Bristol, Bristol, UK, ^4^National Heart and Lung Institute, Imperial College London, UK, ^5^Centre for Statistics in Medicine, University of Oxford, Oxford, UK.*

|  | | ITEM | RECOMMENDATION | Section/ Paragraph |
| --- | --- | --- | --- | --- |
| 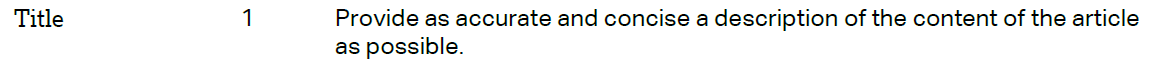 | | | Title/ paragraph 1 |  |
| 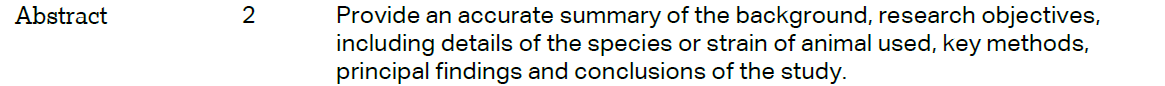 | | | Abstract  Page 2 |  |
| INTRODUCTION | | |  |  |
| 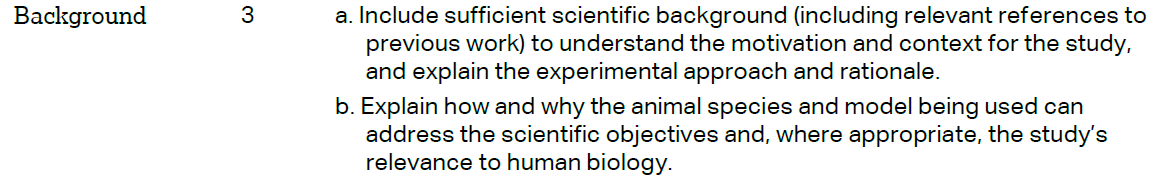 | | | Introduction/paragraph 1 and 2 |  |
| 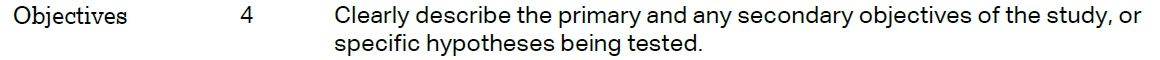 | | | Introduction/paragraph 3 |  |
| METHODS | | |  |  |
| 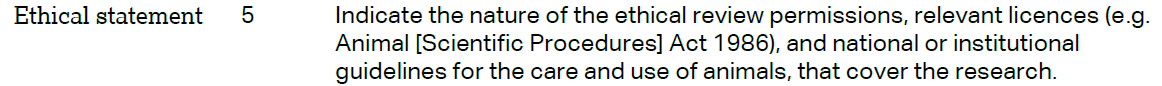 | | | Animals and housing/ paragraph 1  Page 6 |  |
| 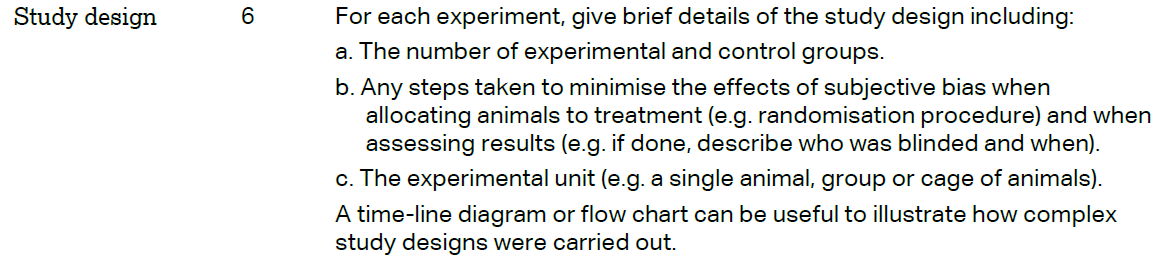 | | | Mouse model/  paragraph 1  Page 7 |  |
| 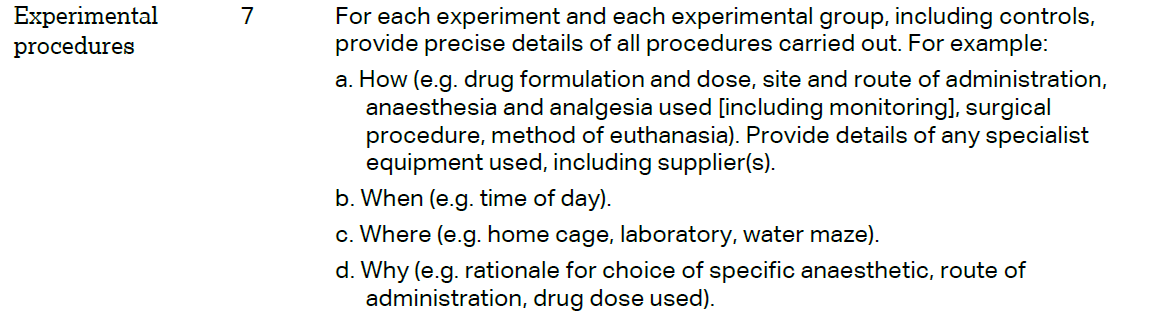 | | | Mouse model/  paragraph 1.  Page 7  Animals and housing/ paragraph 1  Page 6 |  |
| 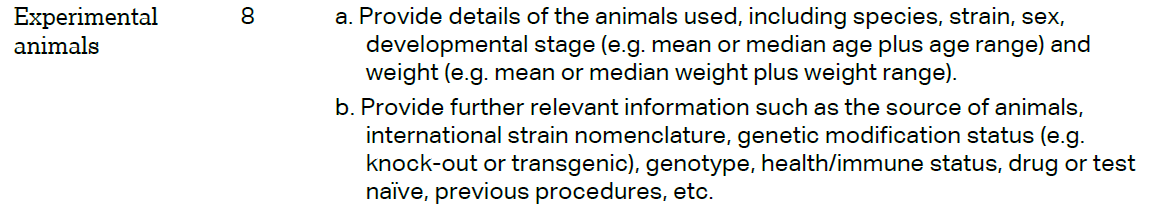 | | | Mouse model/  Paragraph 1  Page 7  Animals and housing/  Paragraph 1  Page 6  Primary Cultures  Paragraph 1  Page 7 |  |

The ARRIVE guidelines. Originally published in *PLoS Biology*, June 2010^1^

| 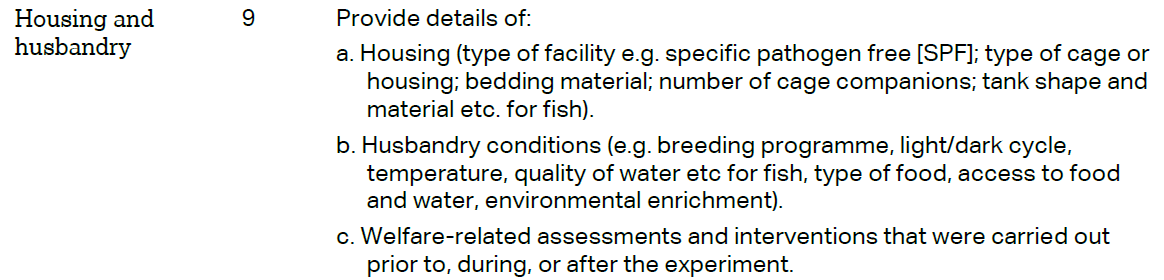 | Animals and housing/ paragraph 1  Page 6 | |
| --- | --- | --- |
| 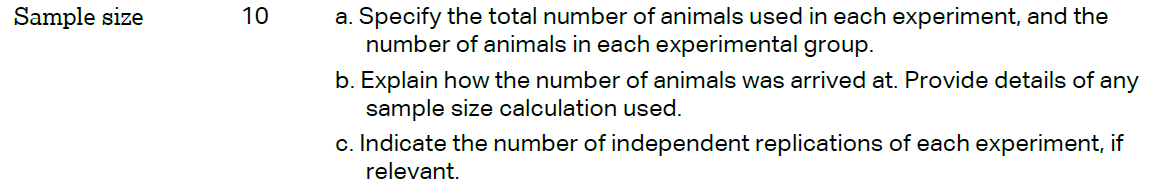 | Animals and housing/ paragraph 1  Page 6  Mouse model/ paragraph  1  Page 7 | |
| 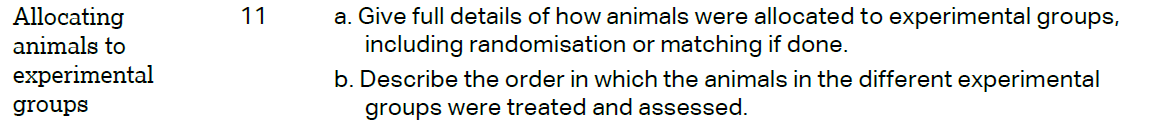 | Mouse model/ paragraph 1  Page 7 | |
| 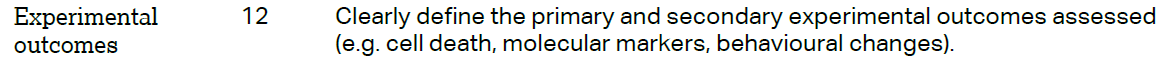 | Animals and housing/ paragraph 1  Page 6 | |
| 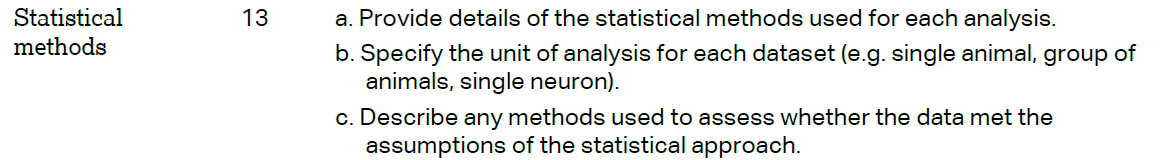 | Statistics/ paragraph 1  Page 10 | |
| RESULTS |  | |
| 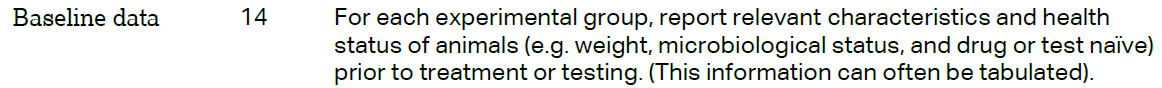 | Materials and methods/ Animals and housing/ paragraph 1  Page 6 | |
| 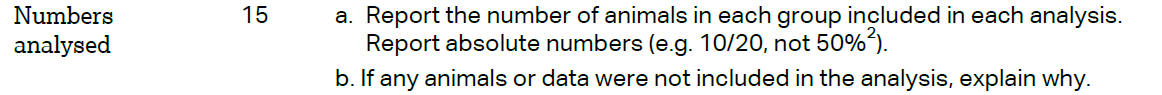 | On each corresponding figure | |
| 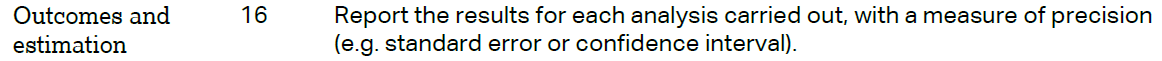 | On each corresponding figure | |
| 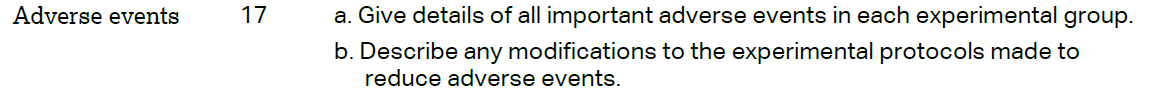 | ---- | |
| DISCUSSION |  | |
| 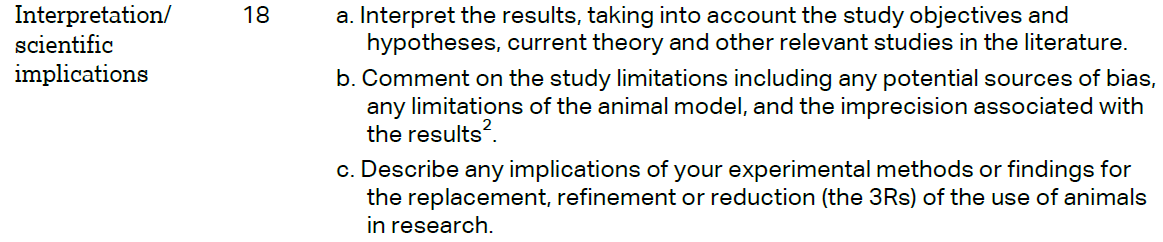 | Discussion/ Pages 15-18 | |
| 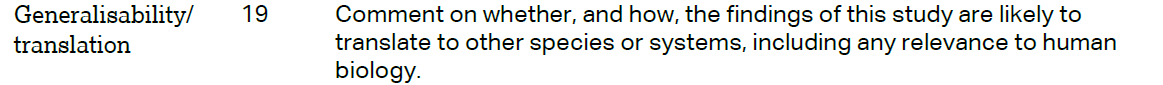 | Discussion/ paragraph 4 | |
| 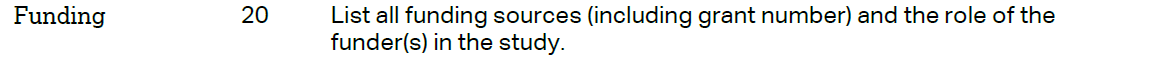 | | Acknowledgments/  paragraph 1 |


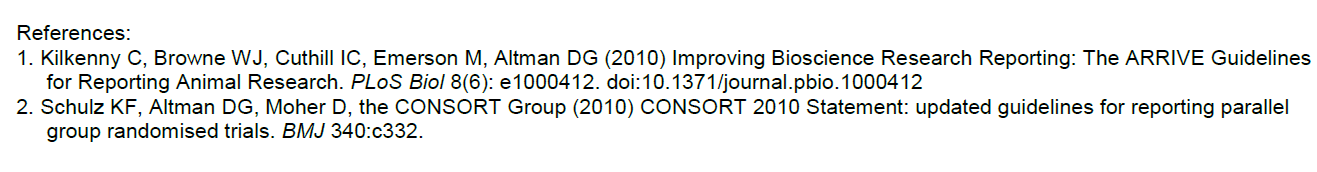

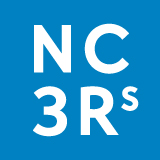

Supplement: S1 File — (DOCX) [file pone.0131879.s001.docx]
